# Alpha Cyano-4-Hydroxy-3-Methoxycinnamic Acid Inhibits Proliferation and Induces Apoptosis in Human Breast Cancer Cells

**DOI:** 10.1371/journal.pone.0072953

**Published:** 2013-09-05

**Authors:** Lamia Hamdan, Zoheir Arrar, Yacoub Al Muataz, Lutfi Suleiman, Claude Négrier, Joseph Kajima Mulengi, Habib Boukerche

**Affiliations:** 1 Unité de Recherche Mixte EA4174, Université Claude Bernard Lyon1, INSERM, Lyon, France; 2 Department de Chimie Organique, Substances Naturelles et Analyses, University de Tlemcen, Tlemcen, Algerie; Enzo Life Sciences, Inc., United States of America

## Abstract

This study investigated the underlying mechanism of 4-hydroxy-3-methoxycinnamic acid (ACCA), on the growth of breast cancer cells and normal immortal epithelial cells, and compared their cytotoxic effects responses. Treatment of breast cancer cells (MCF-7, T47D, and MDA-231) with ACCA resulted in dose- and time-dependent decrease of cell proliferation, viability in colony formation assay, and programmed cell death (apoptosis) with minimal effects on non-tumoral cells. The ability of ACCA to suppress growth in cancer cells not expressing or containing defects in p53 gene indicates a lack of involvement of this critical tumor suppressor element in mediating ACCA-induced growth inhibition. Induction of apoptosis correlated with an increase in Bax protein, an established inducer of programmed cell death, and the ratio of Bax to Bcl-2, an established inhibitor of apoptosis. We also documented the ability of ACCA to inhibit the migration and invasion of MDA-231 cells with ACCA in vitro. Additionally, tumor growth of MDA-231 breast cancer cells in vivo was dramatically affected with ACCA. On the basis of its selective anticancer inhibitory activity on tumor cells, ACCA may represent a promising therapeutic drug that should be further evaluated as a chemotherapeutic agent for human breast cancer.

## Introduction

Breast cancer is one of the most devastating malignant neoplasm’s. Patients frequently already have clinical evidence of tumor dissemination at diagnosis, and many more show local distant recurrent disease shortly after surgical excision of the primary tumor [1]. Curative therapy is not available for these patients and the vast majority of will succumb to disease progression [2], [3]. Given this bleak picture, it is vital to develop new, efficacious, and rationally designed treatment strategies to, ideally, prevent or effectively treat metastasis.

The so called “Warburg effect” describes cancer cells increased use of anaerobic pathways to back up their energetic survival as compared to normal cells [4], [5]. Indeed, 62% of all known cancers show an increased expression of the genes involved in the glycolysis pathway [6]. In this context, targeting metabolic pathways is of particular interest, as drugs that target more specific metabolic points of gycolysis in tumor cells might represent a potentially novel strategy for cancer treatment [7]. The monocarboxylate transporters (MCTs), a conserved protein family that transports lactate and pyruvate through cell membranes [8] has been shown to play an important role in tumor progression [9]. These data suggest that therapeutic strategies that target MCTs may block proliferation and spread of tumor cells. Among inhibitors of MCTs is the α-cyano-4-hydroxycinnamic acid which inhibit tumor growth in vitro through its ability to block lactate efflux [10]–[12]. Recent surveys revealed that α-cyano-4-hydroxycinnamic acid significantly induces necrosis in multiple cancers including, glioblastoma, and tumor of the prostate by increasing lactic acid production and inhibiting plasma membrane MCT activity [13]–[15]. Further studies have shown that MCT inhibitors decreases the size of tumors and sensitizes hypoxic tumor regions to radiotherapy [16]. However, despite evidences suggesting that MCTs inhibitors that target energetic metabolism pathways represent strong candidates for cancer treatment [7], little is known how they affect cancer cell proliferation and viability, thereby causing inhibition of cancer development and progression.

In this study, we investigated the cytotoxicity and anti-proliferation activity of ACCA on breast cancer cells. We document that treatment of breast cancer cells with ACCA inhibited growth and induced apoptosis. We also find that ACCA ca potentially decrease the migration and invasion of MDA-231 cells *in vitro* and dramatically impaired their capacity to form tumors in vivo. Our results suggest that the mechanism of action of ACCA includes direct induction of pro-and anti-apoptotic genes that occurs independent of p53 status in breast cancer cells. Based on these results, we suggest that ACCA may be a candidate for further evaluation as a chemotherapeutic agent for human breast cancer.

## Materials and Methods

### Cell Lines, Reagents, and Culture Media

Immortal human breast epithelial cells line HBL100, and MCF-7, T47D and MDA/MB 231 (MDA-231) human breast adenocarcinoma cell lines were purchased from American Type Culture Collection (Manassas, VA, USA). HBL-100 and MCF-7 (harboring wt p53 ), T47D (harboring mutant p53), and MDA-231 (harboring mutant p53) [17]. were respectively grown in DMEM, MEM and RPMI-1640 medium. They were supplemented with 10% heat inactivated fetal bovine serum (FBS), 1% L-glutamine and 1% penicillin-Streptomycin. MEM medium was supplemented with 0.1 mM non-essential amino acid (NEAA) and 10 µg/ml insulin. All media and supplements were obtained from Life Technologies (Saint Aubin, France). MTT (3-(4,5-dimethyl-2-thiazolyl)-2,5-diphenyl-2H-tetrazolium bromide) and annexin V-PI staining kit were purchased from Calbiochem (Calbiochem, Darmstadlt, Germany).

### 2. The Synthesis of α-cyano-4-hydroxy-3-methoxycinnamic Acid (ACCA)

**Figure 1:**
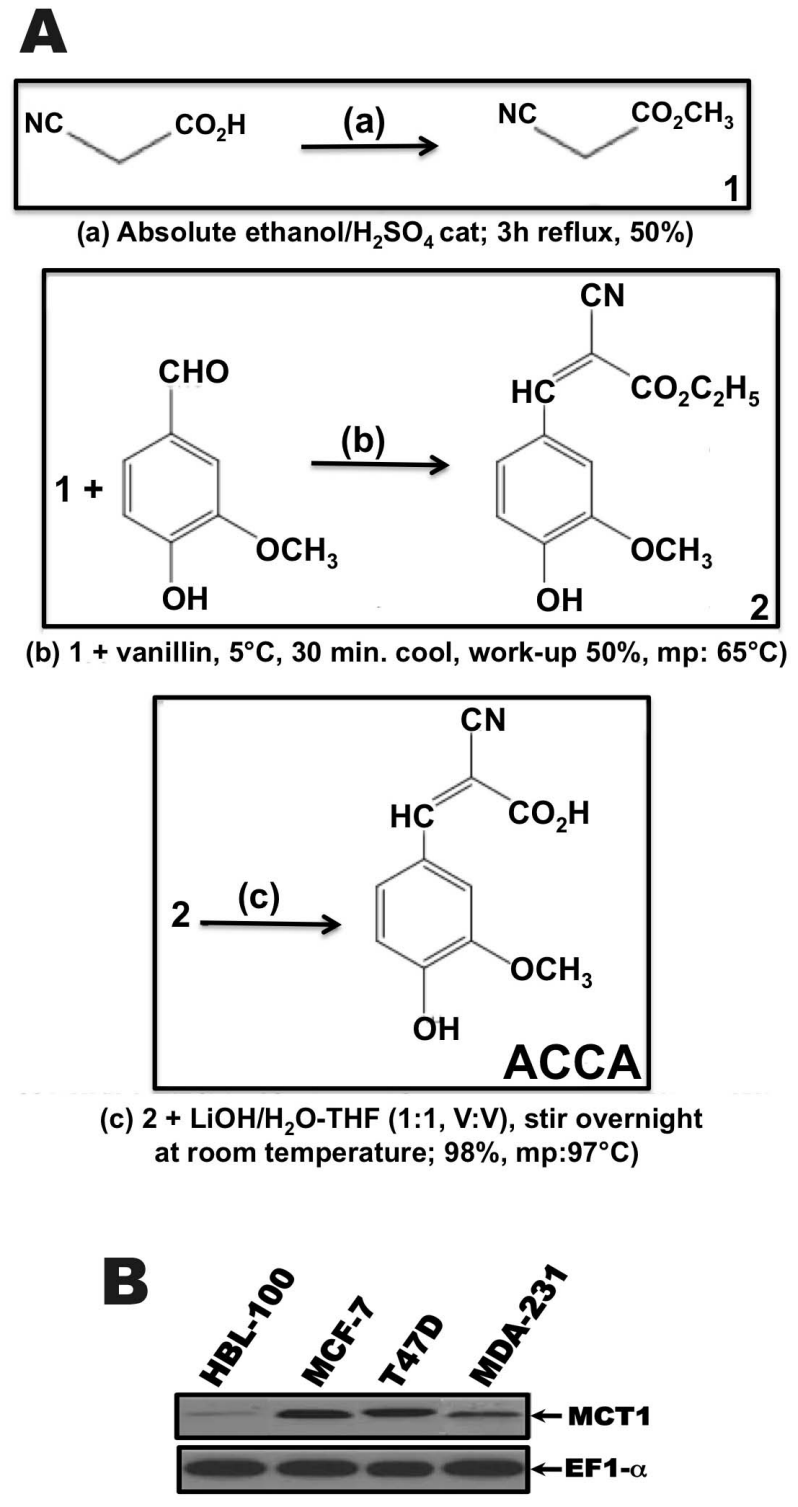
Structure, scheme synthesis of α-Cyano-4-Hydroxy-3-Methoxycinnamic Acid (ACCA) and expression of MCTs in immortal human epithelial cells and human breast cancer cells. (A) Structure and scheme of ACCA synthesis. (B) Expression of MCT1 in immortal normal human breast epithelial cell line, HBL100, and breast cancer cell lines MCF-7, T47D, and MDA-231. Lysates of the indicated cell type were analyzed by western blotting and stained with MCT1 antibody as described in «materials and method». Membranes were reprobed with EF-1α antibody to confirm equal loading.

ACCA was prepared as follow (Fig. 1A)

#### a) Synthesis of ethyl cyanoacetate (solution#1)

Cyanoacetic acid (20.4 g, 0.24moles) was dissolved in 27 ml of absolute ethanol. Then, 0.5 ml of H_2_SO_4_ was slowly added. The resulting solution was refluxed for 3h. Excess alcohol and water was removed at 50°C under diminished pressure. The residue was cooled at room temperature and neutralized with a concentrated aqueous solution of Na_2_CO_3_. The aqueous phase was extracted three times with 40 ml of ether and the combined organic extracts were dried over anhydrous magnesium sulfate. Ether was thoroughly removed under diminished pressure to leave a colorless liquid that was purified by distillation (97–98°C). Yield: 48%. ^1^H NMR (CDCl_3_): 4.16 (d, J = 1Hz, 2H), 3.43 (d, J = 0.94Hz, 2H), 1.21 (s, 3H).

#### b) Synthesis of ethyl α-cyano-4-hydroxy-3-methoxycinnamate (solution#2)

In a dry three-necked flask containing 50 ml of dry ethanol were successively introduced ethyl cyanoacetate (7.4 g, 65 mmol), clear sodium (1 g, 44 mmol) and vanillin (67 g, 44 mmol), keeping the temperature at 5°C. The mixture was stirred until dissolution of sodium was complete. After stirring for further 30 min, 5 ml of glacial acetic acid was added and the reaction was kept overnight at room temperature. Then, 10 ml of distilled water was then carefully added to the cooled solution. The aqueous layer was extracted with 25 ml of ethyl acetate, and the organic extract was dried over MgSO_4_ and concentrated. A yellow solid was obtained. Yield: 61%. Mp: 53°C. ^1^H-NMR (CDCl_3_): 1.27(t, J = 7Hz, 3H), 4.21(q, J = 7Hz, 2H), 3.80(s, 3H), 5.31(s broad, 1H), 6.67(d, J = 1.8Hz, 1H), 7.59(d, J = 1.8Hz, 1H) 7.75(s, 1H), 8.23(s, 1H).

#### c) Alpha-cyano-4-hydroxy-3-methoxy-cinnamic acid (ACCA) (solution#3)

To solution #**2** (10.9 g, 44 mmol) containing 30 ml methanol was carefully added a solution of LiOH (1.1 g, 44 mmol/H_2_0) at 0°C. The reaction was stirred at room temperature overnight. The solvent was removed and the aqueous layer was extracted with 30 ml of ethyl acetate. Then, the extract was washed with an aqueous solution of HCl (pH ≤2) and further extracted three times with 40 ml of ethyl acetate. The combined organic extracts were washed once with brine and then dried over Mg_2_SO_4_. Removal of solvent left a yellow solid that was recrystallized in water/ethanol (9∶1 v/v). Yield: 98%. Mp: 97°C. ^1^H NMR (CDCl_3_): 3.99(s, 3H), 5.75(s, 1H), 6.67(d, J = 1.8Hz, 1H), 7.60(d, J = 1.8Hz, 1H), 7.85 (d, J = 1.8Hz, 1H), 8.16 (d, J = 1.8Hz, 1H), 9.82(s, 1H).

A 2 mM stock solution of ACCA (alpha cyano-4-hydroxy-3-methoxycinnamic acid was prepared in dimethylsulfoxide (DMSO) and stored at −20°C. ACCA was added to cells at the indicated concentrations. The DMSO control concentration was less than 0.1% (v/v). All solutions were filter sterilized using 0.22-µm syringe-filter units. The stock solution was then diluted to the required final concentration in serum-free medium.

### MTT Assay and Assessment of Cell Growth and Viability

The MTT assay is based on the enzymatic reduction of the tetrazolium salt MTT in living, metabolically, active cells and was performed as described [18]. MTT Briefly, cells (1×10^4^/well) were plated in 96-well plates for 24h. At the end of incubation, cells were treated with ACCA at the indicated concentrations for 24 and 48h at 37°C, under a 5% CO_2_ atmosphere. Control cells were treated with 0.1% DMSO. At the end of the incubation, MTT was added to the cells for 3h. at 37°C. Then, 100 µl of SDS 10% was added to dissolve the formazan crystals. Absorbance was measured at 540 nm using a THERMO max microplate reader. Each assay was performed in duplicate. Cell viability was evaluated by assessing trypan blue inclusion/exclusion of isolated cells under ligh microscope and scoring the percentage of cells exhibiting blue staining. Cells (3×10^5^) were seeded into a six-well plate for 24 h. and then treated with 50 µM of ACCA. After 1, 2, 3, 6, and 10 days treatment, floating and attached cells were isolated by trypsination, recovered by centrifugation and mixed with 1∶1 with trypan blue reagent. Cells (∼400) were counted in all four fields of a hemocytometer under a standard slide microscope.

### Colony Formation Assay

Cells growing in log phase were seeded at a density of 1000–2000 cells/well into 60 mm dishes in complete medium. After allowing the cells to adhere for 24 h, medium was replaced with complete medium containing ACCA at the indicated concentrations. Cells were allowed to grow for 3–4 weeks, with a medium change containing ACCA in complete medium, performed every fourth or fifth day. Colonies were then fixed and stained with Methylene blue-50% ethanol and counted. Individual assays were performed in triplicate with a total of three plates per data point.

### Apoptosis Analysis by Flow Cytometry

Tumor cells (10^6^ cells per 60 mm dish) were treated with the indicated concentrations of ACCA for 48h à 37°C. Cells were trypsinized and washed twice with phosphate buffered saline (PBS) and collected by centrifugation at 1000 rpm for 5 minutes at room temperature. Aliquots of cells (5×10^5^/ml) were resuspended in complete media (0.5 ml) and then stained with fluorescein isothiocyanate-labeled Annexin-V kit according to the manufacturer’s instructions. PI (1 µg/ml) was added to the samples after staining with Annexin-V kit to exclude late apoptotic and necrotic cells. Flow cytometry was performed immediately after staining.

### Western Bloy Analysis

After treatments, ice-cold PBS solution was used two times to rinse cells. Cells were then lysed with cell lysis buffer [Tris (pH 7.5), 50 mM; EGTA 5 mM; NaCl 120 mM; α-glycerophosphate 20 mM; Nonidet P-40 1%; Na pyrophosphate 15 mm; Na fluoride 50 mM; Na orthovanadate 10 mM; phenylmethylsulfonyl fluoride 0.5 mM; aprotinin 10 µg/ml; leupeptin 10 µg/ml; glycerol 20%] and dishes incubated for 10–30 min at 4°C. Cells were scraped into lysis buffer, and lysates were clarified by centrifugation (12,000 rpm, 15 min at 4°C). Protein concentrations was determined using a kit from Bio-Rad and western blot analyses were performed as previously described [19]. Aliquots (50 ug) were solubilized in Laemmli buffer, separated by SDS-PAGE, and transferred to nitrocellulose membranes. Membranes were blocked 2 hours at 4°C in TBST (5% nonfat milk in 10 mM Tris/HCl, 100 mM NaCl, and 0.1% Tween-20, pH 7.6). Membranes were exposed to the primary antibodies, followed by washing (3×15 min. with TBST), and antibody bound proteins were detected by enhanced chemiluminescence reagents (Pierce), according to the manufacturer’s protocol. The following antibodies were used: rabbit anti-MCT1, mouse anti-Bax, rabbit anti-Bcl-2 (Santa-Cruz Biotechnologies, CA), and anti-EF-1α (Millipore, Billerica, MA).

### Cell Invasion, Migration and in Vivo Tumorigenicity Assays

Transwell invasion and migration assays were performed as described [19] in a modified Boyden chamber (BD Bioscience, Bedford, MA, USA). Briefly, the Matrigel was allowed to rehydrate for 2 h at room temperature by adding warm, serum-free DMEM. The wells of the lower chamber were filled with DMEM containing 10% NuSerum, and the chambers were each assembled by placing the uncoated membrane between the lower and upper compartments according to the manufacturer’s instructions. MDA-231 cells (10^5^) were seeded in the upper compartment in serum-free DMEM containg 0.1% BSA. ACCA was then added at the indicated concentrations to the upper chambers and incubated for 48 h at 37°C in a 5% CO2 humified incubator. For migration, cells (5×10^4^) were added to the upper compartments of the BioCoat chambers supplemented with the indicated concentrations of ACCA. The wells of the lower chamber were filled with RPMI 1640 containing 10% NuSerum, and the chambers were each assembled similarly to the method described above for migration assay. The migration assay was carried out at 37°C for 15–18 h at 37°C. Cells that invaded the Matrigel or migrated to the underside of the coated membrane were fixed, stained with the RAL 555 staining kit. Stained cells were counted and normalized relative to the number of seeded cells. Experiments were assayed in triplicate, and at least 10 fields were counted in each experiment. In vivo tumorigenicity assay was performed as described [19]. Briefly, exponentially growing cells MDA-231 cells were suspended in PBS and mixed in a 1∶1 ration with Matrigel. Then, 100 hundred ul of cells (2×10^6^) were inoculated s.c. on the right flank of each nude mouse above the hind limb. One week after tumor inoculation, the mice were treated with vehicle or ACCA (25 µmol in 200 µl; ref. [21]) five times/week begining one week after subcutaneous tumor cell injection. Tumor growth rate was determined as described [20].

### Data Analysis

For cell proliferation/MTT assays, data are expressed as a mean±SEM of multiple experiments, with each experiment including three to six determinations, or alternatively data are presented as mean±SEM of OD. Statistical significance was determined using Student’s t test.

## Results

### ACCA Inhibits Proliferation of Human Breast Cancer Cells in a Dose and Time-Dependent Manner

**Figure 2:**
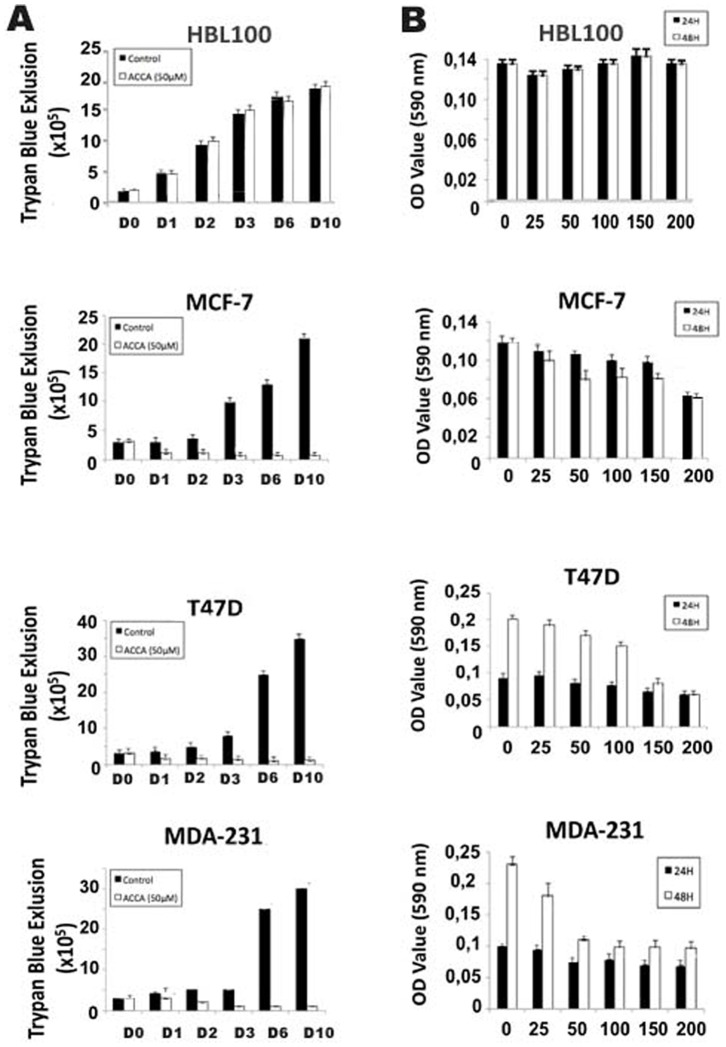
Effect of ACCA on the in vitro growth and proliferation rate of immortal human epithelial cells and human breast cancer cells. (A) The indicated cell type were either untreated (control) or treated with 50 uM of ACCA and cell growth was assessed for 1, 2, 3, 6, and 10 days. The trypan blue exclusion test is used to determine the number of viable cells. Values represent number of cells x10^5^. (B) The indicated cell type either untreated or treated with different doses of ACCA were seeded in 96 wells, and a standard MTT viability test was performed 24h and 48h. postreatment as described in «materials and methods». Columns, mean±SD, n = 3.

Because ACCA is a known inhibitor of monocarboxylate transporters (MCTs) with about a tenfold higher afficacy for MCT1 as compared with others MCTs [8], we first determined by western blot analysis the expression of the MCT1 protein in immortal normal human breast epithelial cells, HBL100, and breast cancer lines, including MCF-7, MDA-231, and T47D. Consistent with previous studies [22], we found that MCT1 protein is elevated in all three breast cell lines compared to HBL-100 immortal breast cell line (Fig. 1B). We next investigated the effect of ACCA on cell viability. MCF-7, MDA-231, and T47D breast cancer cells were treated with vehicle or 50 µM of ACCA for different time intervals and cell viability was evaluated by trypan blue dye exclusion method. A shown in Fig. 2A, ACCA exhibited a significant reduction in cell viability across MCF-7, MDA-231 and T47D cell lines treated with 50 µM of ACCA for 1, 2, 3, 6 or 10 days as compared to control cells. In contrast to malignant breast tumor cells, treatment of immortal normal human breast epithelial cells, HBL-100, with vehicle or the same concentration of ACCA did not influence the cell growth of HBL-100 cells (Fig. 2A). To confirm these data, MCF-7, MDA-231, and T47D breast cancer cells were treated with various concentrations of ACCA, ranging from 25 to 200 µmol/L for 24 and 48h. and cell growth was evaluated by MTT reduction assay. As shown in Fig. 2B, there is no significant difference in growth inhibition in all tumor cell lines treated with a 25 µM ACCA for 24h compared with untreated cells. However, and consistent with the data obtained above, on treatment of cells for 48 h. with increasing concentrations of ACA (25, 50, 100, 150 and 200 µM), tumor cell growth was strongly inhibited in a dose-dependent manner (Fig. 2B). This suppressive effect of ACCA on tumor cells was most remarkable at the concentration of 200 µM, 150 µM and 50 µM for MCF-7 (54.47%; *P*<0.005), T47D (77.47%; *P*<0.005), and MDA-231 (64.67%; *P*<0.005) cells, respectively. In contrast, treatment of HBL100 immortal breast cell line, with increasing concentrations of ACCA (25, 50, 100, 150 and 200 µM), do not affect the growth of MDA-231, T47D and MCF-7 cells (Fig. 2B). In total, these results suggest that ACCA acting through MCT1 selectively inhibits the growth of breast cancer cells in vitro.

We next questioned whether the growth-inhibitory effect of breast cancer cell proliferation persists on removal of ACCA. MDA-231 cells were treated with medium alone or containing 50 µM of ACCA for 48 h., and proliferation was assessed by MTT assay (d0). Our data show that, consistent with our previous results, ACCA significantly inhibited tumor cell proliferation (d0, OD control, 0.235; OD ACCA, 0.084, 62.38% inhibition, *P*<0.001). Plating medium was removed and replaced with growth medium containing 10% of FBS, and cell growth was monitored for a further 4 days. Our data show that the suppressive effects of ACCA at day 4 (d4) were similar to that observed at d0, (d0, OD control, 1.775; OD ACCA, 0.580, 67.33% inhibition, *P*<0.001). These results clearly demonstrate that the inhibitory effect of ACCA on cell proliferation persists, even on removal of the drug.

### ACCA Blocks the Clonogenic Ability of Breast Cancer Cells

**Figure 3:**
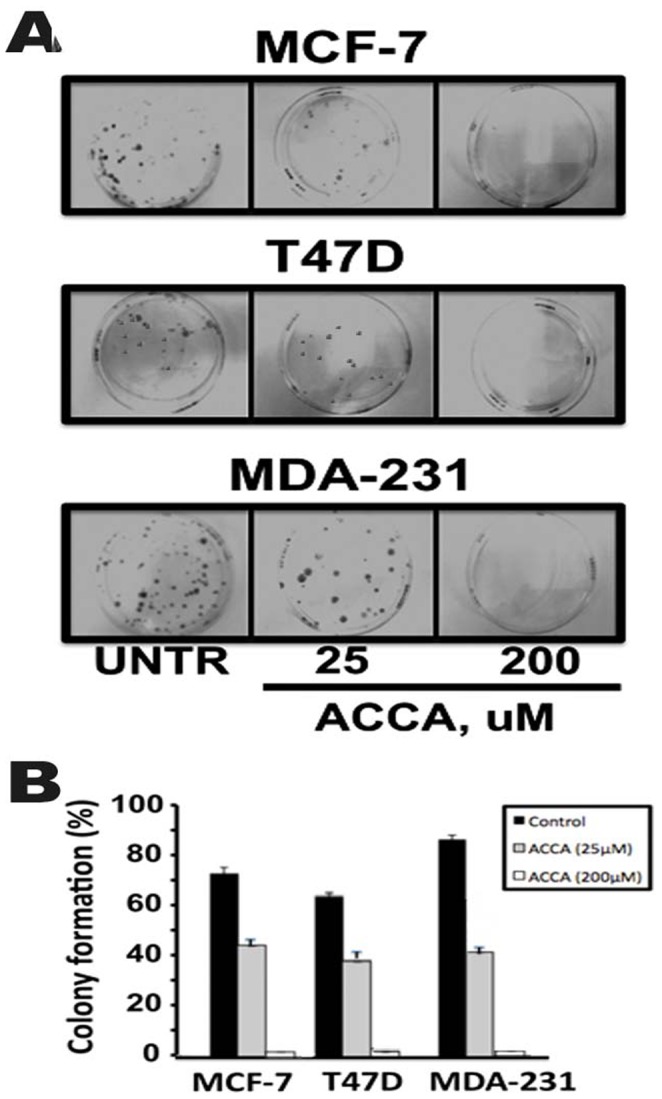
Effect of ACCA on colony formation of human breast cancer cells. The indicated cell type either untreated (UNTR) or treated with 25 or 200 uM of ACCA were allowed to grow for 3–4 weeks and the number of colonies formed was measured as «described in materials and methods». (A) Representative images of the cloning wells (A) and quantification (B) are shown. Columns, mean±SD, n = 3.

We next investigated whether ACCA had any effect on the ability of breast cancer cells to form colonies. Cells were seeded (1000–2000 cells/well) in complete medium in six-well plates and allowed to adhere for 24 h. The medium was then replaced with complete medium containing ACCA (0–25–200 µM), and the ability of MCF-7, T47D and MDA-231 cells to form colonies was monitored over the next 21 days. Our results show that in the absence of any ACCA, MCF-7, T47D and MDA-231 cells cells have a robust ability to form colonies and that ACCA inhibits this in a dose-dependent manner in all three tumor cell lines (Fig. 3A and 3B). A significant inhibitory effect of ACCA on breast cancer cells colony formation is apparent at even 25 µM doses of ACCA and with a 200 µM dose, the ability of breast cancer cells to form colonies is completely blocked. These results extend our previous observations indicating that that ACCA caused inhibition of cell death at a concentration greater than 25 µM and suggest that ACCA is a potent suppressor of cell growth.

### Induction of Apoptosis in Human Breast Cancer Cell Lines Correlates with an Elevation in Bax Levels

We next determined whether ACCA decreases cell viability of breast cancer cells through the induction of apoptosis. Thus, cells were treated for 48h with 200 uM of ACCA and phosphatidylserine translocation was measured by flow cytometry and annexin V labeled with FITC. Annexin V, a Ca2^+^-dependent phosphatidylserine binding protein, detects phosphatidylserine translocation onto the outer plasma membrane leaflet. Propidium iodide (PI) staining was used in conjunction with Annexin V-FITC for the detection of necrotic cells.

**Figure 4:**
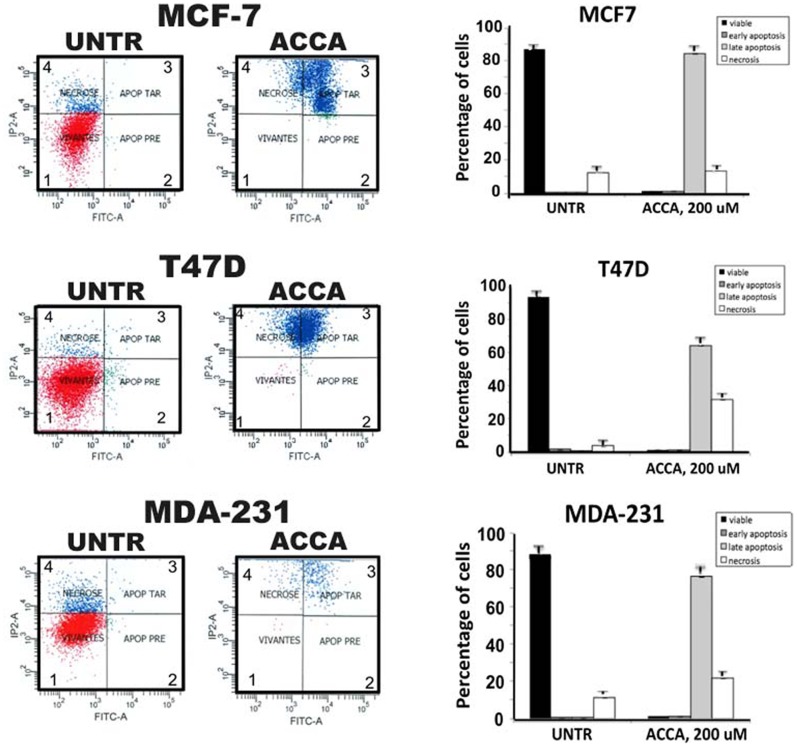
Effect of ACCA in breast cancer cells. The indicated cell type either untreated (UNTR) or treated with 200 uM of ACCA for 48h. were stained with FITC-labeled Annexin-V and propidium iodide (PI) and immediately analysed by flow cytometry. (A) Data from a representative of four experiments are shown. Cells in the bottom left quadrant 1 represent viable cells (low Annexin V and PI staining, AnV^−/^PI^-^); cells in the the bottom right quadrant 2 represent early apoptotic cells (high annexin V staining but low PI staining, AnV^+^/PI^-^);); cells in the top right quadrant 3 represent late apoptotic cells (low annexin V and high PI staining, AnV^−/^PI^+^);), and cells in the top right quadrant 4 represent necrotic cells (high annexin V and PI staining, AnV^+^/PI^+^). Shown are representative data from one of three independent experiments with samples in triplicate. (B) The percentage of early apoptotic cells (only Annexin-V stained), late apoptotic and necrotic cells was calculated using the CellQuest software (Becton Dickinson, San Jose, CA, USA). Columns, mean±SD, n = 3.

**Figure 5:**
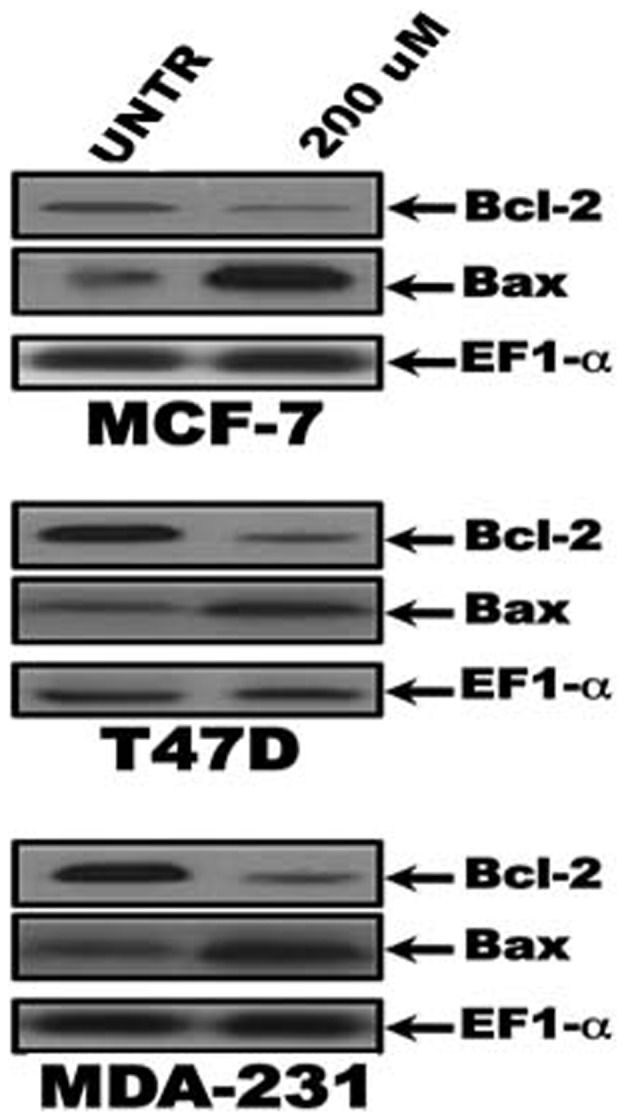
Effect of ACCA on the levels of Bcl-2 family proteins in breast cancer cells. Lysates of the indicated cell type either untreated (UNTR) or treated with 200 uM of ACCA for 48h. were analyzed by western blotting and stained with Bcl-2 and Bax antibodies as described in «materials and method». Membranes were reprobed with EF-1α antibody to confirm equal loading.

Annexin V-negative/PI-negative, Annexin V positive/PI-negative, Annexin V positive/PIpositive, AnnexinV-negative/PI-positive cells represent the viable cells, the cells in early apoptosis, late apoptosis, and necrosis, respectively. As shown in Fig. 4, ACCA induced early and late apoptosis in 85.8, 77.6 and 65.9% of the cell population of MCF-7, MDA/MB 231 and T47D respectively at dose of 200 µmol/L for 48 h. compared to untreated cells. We also found that ACCA induces necrosis in 21.6, 13, 31.8% of the cell population of MCF-7, MDA/MB 231 and T47D respectively, suggesting that ACCA might also cause significant cellular injury and increased apoptotic cell death (Table 1). We next define potential target genes associated with apoptosis that might be regulated by ACCA, thereby resulting in apoptosis in breast cancer cells. Because of the importance of Bcl-2 family proteins in the regulation of apoptosis, we monitored the levels of expression of antiapoptotic proteins (Bcl- 2) and the proapoptotic proteins (Bax) in breast cancer cells. As shown in Fig. 5, ACCA decreased Bcl-2 protein level (1.8-, 2.4-, and 2.6-fold, respectively) whereas Bax protein expression were elevated (3.4-, 2.4, and 3.2-fold, respectively) in MCF-7, T47D and MDA- 231 breast cancer cells, respectively. In contrast, the levels of the various pro- and antiapoptotic proteins did not change significantly in HBL-100 cells following treatment with.

**Table 1:** Effect of ACCA on the induction of apoptosis and necrosis in breast cancer cells.

ACCA concentrations (µM)		% of Cells			
		Viable	Early apoptosis	Late apoptosis	Necrosis
**MCF-7** b	C^a^	87,4	0,3	0,3	11,9
	200	1,2	1,3	84,5	13
**T47D** b	C^a^	94,4	1,5	0,6	3,5
	200	1,4	1,3	64,6	31,8
**MDA-231** b	C^a^	87,9	0,4	0,4	11,3
	200	1,1	1,3	76,3	21,6

a C = control group.

b Cells were analyzed by flow cytometry after being stained with Annexin V-FITC and PI. The % of cells were calculated using CELL Quest software.

ACCA (data not shown). These studies suggest that ACCA increases the ratio of of pro- to antiapoptotic proteins, thereby tipping the balance of cancer cells from survival to programmed cell death.

### ACCA Treatment Decreases Cell Motility, Invasion and in Vivo Tumor Growth of MDA-231 Human Breast Cancer Cells

**Figure 6:**
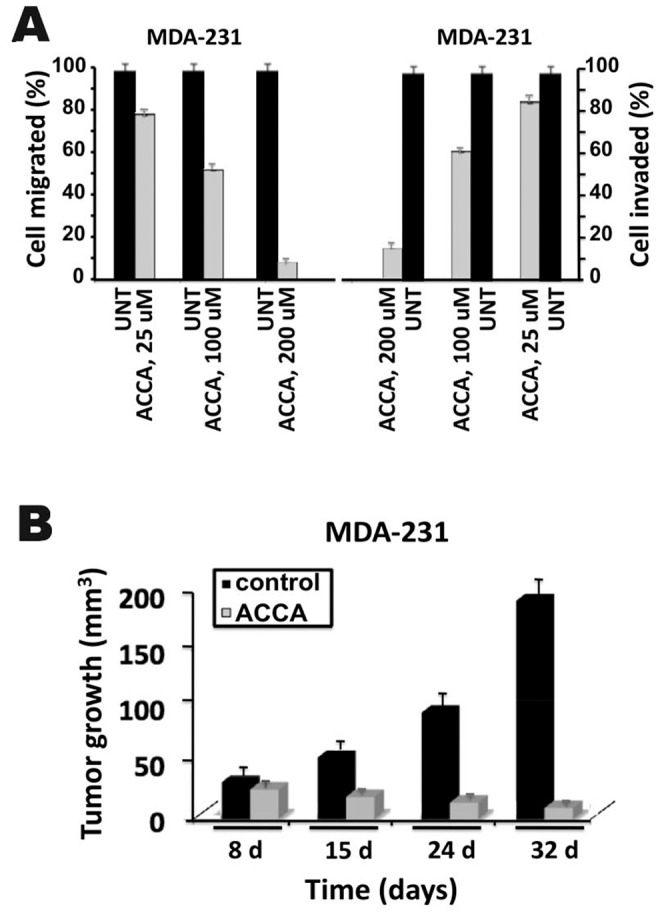
Effect of ACCA migration and invasion in vitro, and breast cancer tumor growth in vivo. (A) For invasion, MDA-231 cells (10^5^) were seeded in the upper compartment of a Matrigel chamber in serum-free DMEM containing 0.1% BSA and allowed to invade for 48 h at 37°C in the presence of the indicated concentrations of ACCA. The lower compartment contained 0.5 ml of DMEM and 10% NuSerum. For migration, MDA-231 cells (5×10^4^) were seeded in the upper compartment in serum-free DMEM containg 0.1% BSA and allowed to migrate for 15–18 h at 37°C in the presence of the indicated concentrations of ACCA. The lower compartment contained 0.5 ml of DMEM and 10% NuSerum. At the end of the invasion and migration assays, the filters were removed, fixed, and stained as indicated in “Materials and Methods.” Invasion and migration were determined by counting of the cells that had migrated to the lower side of the filter with a microscope. Ten fields/cell line were counted. *Bars*, SD of triplicate samples from three independent experiments. The migration and invasion of control cells was used as a positive control (set at 100%). «UNT» refers to untreated cells. (B) MDA-231 cells (2×10^6^) mixed in a 1∶1 ration with Matrigel were inoculated s.c. on the right flank of each nude mouse above the hind limb. One week after tumor inoculation, the mice were treated five times/week begining one week after subcutaneous tumor cell injection with vehicle (control) or ACCA (25 µmol in 200 µl). Tumor sizes at different times are expressed as the mean of the sum of two perpendicular diameters. *Bars*, SD of tumor volumes from five mice.

To investigate whether ACCA can affect important biological properties of breast cancer cells, we first used in vitro cell migration and invasion assays. As shown in Fig. 6A, the migratory and invasive ability of MDA-231 cells was significantly affected in a concentration-dependent manner by ACCA. At the highest concentration of 200 uM, ACCA causes an up to ∼90 and 85% reduction in cell migration and invasion, respectively, as compared to untreated cells. We also determine the effect of multiple i.p. injections of ACCA on growth of MDA-231 cells subcutaneously implanted in nude mice. The ACCA concentrations delivered as a treatment protocol in the present study are within the range used in previous animal studies [21]. A shown in Fig. 6B, the average tumor volume in mice treated with ACCA was significantly lower compared to vehicle-treated control mice. At 32 days after tumor cell injection, MDA-231 cells gave rise to small tumors as compared to vehicle-treated control mice (195±12 mm^3^ vs 12±2 mm^3^) indicating that ACCA reduced tumour growth by ∼94%. Although, treatment of mice receiving MDA-231 cells with ACCA at 8 days resulted in a less dramatic decrease in tumor growth, the difference was still evident (Fig. 6B). Of note, animals remained well throughout the entire experiment and no weight loss was observed upon treatment, suggesting that ACCA was well tolerated at this dose regimen (data not shown).

## Discussion

Although treatment of breast cancer has improved considerably, curative therapy is not available for these patients. Unfortunately, the treatments in use today, such as surgery, radiation and chemotherapy, are exhaustive to the patients [2], [3]. There’s therefore great need to focus on new treatments that are effective in fighting specifically the tumors. Breast cancer, as almost malignant tumors, harbors aberrant metabolic pathways which support rapid cell proliferation and provide a growth advantage over normal cells [5]. Previous finding suggest that lactate, the end of product of glycolysis, is produced in large amount in tumors and is more effective than glucose in the tumor cell energy metabolism under aerobic conditions [11], [12], [14], [23]. The mechanism set forward involves a competition between lactate and glucose metabolism for the intracellular NAD^+^pool [24]. In this context, it was shown that the main mechanism underlying this metabolic change include important cytotoxic and cytostatic effects on several human tumors including breast carcinomas [25], [26]. Additionnally, the loss of the reserve capacity of the mitochondrial function in breast cancer cells often leads to cell death via mitochondrial apoptotic signaling pathways [27]. Because MCTs are key players in this process, disruption or alteration of the metabolism of tumor cells via inhibition of MCTs function represent promising therapeutic perspectives for the development of targeted therapies in breast cancer [9].

In this study, we document the antibreast carcinoma and apoptosis-promoting properties of ACCA. On the basis of the previously confirmed genetic defects in the human tumor cell lines analyzed, including mutations p53 [28], it is evident that the antineoplastic effect of ACCA, acting through MCT1, does not depend on the action of this tumor suppressor gene that is frequently altered in human cancers. The capacity of ACCA to efficiently inhibit the growth of wild-type and mutant p53 breast carcinoma cells, supports the possibility that ACCA may prove efficacious in the treatment of human breast and other cancers. Additionally, it is important to note that the cell viability and proliferation in immortal normal human breast epithelial cells were not significantly altered by ACCA at concentrations ranging from 25 to 200 uM, which significantly induced cell death in breast carcinoma cells. Furthermore, our results showed that ACCA significantly inhibited both migration, invasion and in vivo tumor growth of MDA-231 cells. These data together with previous studies demonstrating similar anti-proliferative effects of α-cyano-4-hydroxycinnamate through inhibition of lactate transporters in other cellular contexts [13]–[15], suggest that ACCA may possess potential therapeutic activity against breast cancer cells, without significantly affecting viability of normal cells.

The Bcl-2 gene family members are important genetic elements in maintaining homeostasis between survival and cell death [29]. Bcl-2 and Bcl-XL bind to the outer membrane of mitochondria and block cytochrome C efflux. In contrast, following induction of apoptosis, Bax translocates from the cytosol to the mitochondria where it enhances release of cytochrome C through the outer membrane of mitochondria. A number of cytotoxic anticancer drugs or apoptotic stimuli has been shown to trigger cytochrome C release through down-regulation of Bcl-2/Bcl-XL and/or up-regulation of Bax [30]. The present study demonstrates that ACCA is capable of inducing apoptosis in breast cancer cells. Cell death triggered by ACCA is accompanied by up-regulation of the proapoptotic protein Bax whereas levels of Bcl-2 significantly decreased. Although previous reports have shown induction of apoptosis in several cancer cell lines [13]–[16], our data are the first to highlight mechanisms by which ACCA induces apoptosis in breast cancer cells. Our investigation also revealed that although Bax expression is regulated positively by wild-type p53 [31], ACCA is able to induce Bax irrespective of p53 status in breast cancer cells, suggesting that alternative pathways can be involved in Bax up-regulation after treatment with ACCA. Our results add to the growing evidence that has been accumulated over the past few years supporting the existence of p53-independent cell death induced by chemotherapeutic drugs [32]–[35]. At present, it unknown whether ACCA directly or indirectly induces Bax expression in breast cancer cells.

In summary, our study demonstrates that ACCA inhibits both breast cancer cell migration/invasion and tumors in vivo, and of induces growth suppression and apoptosis in human breast cancer cell lines containing both normal and mutated p53. The cytotoxicity of this compound is highly related to the expression of apoptotic regulator molecules, such as Bcl-2 and Bax. Of potential importance and warranting expanded studies is the finding that ACCA is more growth inhibitory toward breast cancer and than normal cells. In this context, ACCA may have a great potential to selectively compromise tumor cell viability and to improve the effectiveness of chemotherapeutic agents against breast cancer.
